# A neuro-computational framework for modeling the development of cross-sensory interactions in Autism: from mechanistic understanding to targeted intervention

**DOI:** 10.21203/rs.3.rs-8583228/v1

**Published:** 2026-01-14

**Authors:** Melissa Monti, Sophie Molholm, John J. Foxe, Cristiano Cuppini

**Affiliations:** 1 Department of Electrical, Electronic, and Information Engineering Guglielmo Marconi, University of Bologna, Bologna, Italy; 2 Microtechnology for Neuroelectronics Unit (NetS^3^ lab), Fondazione Istituto Italiano di Tecnologia, Genova, Italy; 3 The Cognitive Neurophysiology Laboratory, Departments of Pediatrics and Neuroscience, Albert Einstein College of Medicine, Bronx, New York, USA; 4 The Frederick J. and Marion A. Schindler Cognitive Neurophysiology Laboratory, The Del Monte Institute for Neuroscience, Department of Neuroscience, University of Rochester School of Medicine and Dentistry, Rochester, New York, USA

**Keywords:** neurocomputational modeling, Hebbian learning, multisensory integration, developmental disorders, autism spectrum disorder

## Abstract

Recent studies have shown that multisensory processing in children shifts from a competitive to a facilitative state as development progresses, and this transition appears delayed in children with autism spectrum diagnosis (ASD). The neural mechanisms underlying this developmental change, and its alteration in ASD, remain largely unknown. To address this gap, we investigated how sensory modalities interact in the developing brain using a biologically plausible neurocomputational model governed by Hebbian learning rules. We also explored the neural substrates that may underlie atypical multisensory development in ASD. Our results suggest that inhibitory cross-modal projections gradually become excitatory during development, mediating the observed shift from competition to facilitation in typical children. Furthermore, our simulations show that the delayed transition in ASD may stem either from reduced neural plasticity or diminished multisensory experience. Our model informs the potential benefits that a multisensory rehabilitation strategy may have on the development of perceptual abilities of ASD children, and possibly on the core social symptoms characterizing Autism as well. By linking computational modelling with behavioural findings, our work provides a framework for understanding atypical sensory development. We anticipate that this model could inform future neurophysiological studies and guide the design of multisensory-based therapeutic interventions for ASD.

## Introduction

We live in a multisensory environment, rich in sensory information coming from different events. To take advantage of such sensory richness and avoid misperception and misinterpretation, the brain must learn how to disambiguate inputs coming from the same events from stimuli belonging to different events. Combining information from multiple sensory modalities (such as vision, hearing, touch, taste, and smell) allows the brain to construct a more comprehensive and accurate representation of the external world. This can lead to improved perception, faster reactions, increased sensitivity, and more adaptive behavior. All of these contribute to a richer and more effective interaction with the surrounding world. A task that has frequently been employed to investigate multisensory gain is the bisensory reaction time task, where responses to multisensory stimuli are expected to be faster compared to responses to unisensory stimuli, thanks to multisensory integrative processes.

Several experimental findings in both humans and animals indicate that multisensory processes are immature at birth and develop progressively during postnatal life. This maturation critically depends on the specific sensory experiences involving cross-modal cues (Bahrick & Lickliter, 2000; Carriere et al., 2007; Ross et al., 2011; Rowland et al., 2014; Santapuram et al., 2022; Wallace, 2004; Wallace et al., 2004; Wallace & Stein, 1997, 2007; Xu et al., 2014; Yu et al., 2010). For example, studies that have systematically altered or compromised early sensory cues (for example by rearing animals in darkness or omnidirectional masking sound) found a correlation between the emergence of integrative abilities with the specific sensory experience perceived during the maturation process (Carriere et al., 2007; Wallace et al., 2004; Xu et al., 2014; Yu et al., 2010). Since development is such a critical period for the full maturation of integrative abilities, it is not surprising that children with neurodevelopmental disorders do not benefit from cross-modal stimuli in the same way as typically developing (TD) children do. In particular, impaired multisensory processing has been repeatedly reported in children with autism spectrum disorders (ASD) (Beker et al., 2018; Brandwein et al., 2013; Crosse et al., 2022; Cuppini et al., 2017; Foss-Feig et al., 2010; Foxe et al., 2015; Ross et al., 2015; Russo et al., 2010; Stevenson et al., 2012; Stevenson, Segers, et al., 2014; Stevenson, Siemann, Schneider, et al., 2014; Stevenson, Siemann, Woynaroski, et al., 2014; Taylor et al., 2010; Wallace & Stevenson, 2014). What is most intriguing about the multisensory deficits observed in the ASD population is that these deficits are clearly noticeable in children, but appear to resolve in adolescents on the spectrum. This is especially true for tasks involving multisensory speech stimuli. Recently, Crosse et al. (2022) used a bisensory reaction time task (in which auditory and visual stimuli are presented alone or together with an inter-stimulus-interval randomly varying between 1 and 3 s) and extended these results to low-level (i.e., non-social) cross-modal stimuli. Notably, this recovery occurs later than that observed for speech stimuli: while multisensory speech integration deficits appear to resolve around 12–13 years of age (Foxe et al., 2015; Ross et al., 2015), integration of simple non-social audiovisual stimuli (e.g., tone pips and visual flashes) remains impaired through adolescence and tends to normalize only in adulthood (18 years and older; Crosse et al., 2022). It is believed that this delayed maturation of multisensory function in ASD stems, at least in part, from the altered cross-modal experiences of ASD children. Indeed, individuals with ASD often show enhanced local processing and reduced global integration, consistent with the “weak central coherence” hypothesis (Frith, 1989; Happé & Frith, 2006). As a result, ASD individuals’ attention may be biased toward a single unimodal component of a cross-modal cue, thereby reducing cross-modal coactivation and limiting the sensory experiences necessary for MSI maturation. However, as they enter puberty and adolescence, increased engagement in social contexts and/or targeted interventions may help to overcome this attentional bias, allowing for more frequent multisensory exposure and consequently promoting the recovery of MSI functions (Foxe et al., 2015; Santapuram et al., 2022). Here, we clarified the neural bases for the delayed maturation of cross-modal interactions in children with ASD. To achieve this, we expanded upon a computational framework previously developed by our research group (Cuppini et al., 2020; Monti et al., 2025) to model the neural mechanisms likely involved in the processing of cross-modal stimuli in the temporal domain (during a bisensory reaction time task), in ASD and TD children as well as TD adults.

Crosse and colleagues also suggested that multisensory experience leads to a shift from cross-modal competition to cross-modal facilitation over time, both in TD individuals and those with ASD. At birth, sensory modalities interact competitively, and it is only later that the typical cross-modal facilitation seen in adults develops. This is in accordance with previous computational efforts and neurophysiological studies (Monti et al., 2025; Yu et al., 2019). It is interesting that this developmental trajectory seems to be delayed in the autistic brain. Thus, we asked: 1) whether our modeling approach could predict the shift from a competitive multisensory processing to one of a facilitative nature, and 2) whether it could explain the neural mechanisms responsible for the delayed transition in ASD individuals.

The bisensory reaction time task implemented in Crosse et al. (2022) requires participants to rapidly switch from processing one sensory modality to another one, thus it can be thought of as a task-switching paradigm (see also Shaw et al., 2020). In particular, the authors analyzed the so-called Modality Switch Effect (MSE), or Switch Cost, which is the additional processing load for the brain when it switches from one sensory modality to a different one. A recent computational study from our laboratory (Cuppini et al., 2020) examined the neural mechanisms underlying the Switch Cost in the healthy adult population. This study found that inhibitory feedback from higher-order brain areas to unisensory regions, characterized by slow dynamics, could drive the interplay between multisensory integration and task-switching, resulting in the MSE. Crosse et al. also analyzed the development of MSE, reporting that it appears to increase with age. Here we tried to unveil the neural underpinnings of age-related changes in the Switch Cost, demonstrating that they could be explained by the evolution of the inhibitory feedback process previously described.

Finally, the identification of the mechanisms contributing to delayed maturation in ASD prompted us to utilize the model as a translational neuroscience framework for predicting the potential effect of rehabilitation strategies. The delay in the full maturation of integrative abilities observed in ASD opens the door to possible perceptual multisensory learning interventions (Beker et al., 2018; Feldman et al., 2023). Therefore, we chose to use our model to test whether providing individuals with ASD with extensive and consistent exposure to multisensory stimuli, which compensates for their altered and reduced cross-modal experience, could accelerate the amelioration of their multisensory functions. We also hypothesized that this should hold true if multisensory training is applied at a young age, within that timeframe when brain structures and mechanisms are more plastic and their development is more sensitive to experience.

## Methods

In the following section, the model architecture and the implemented mechanisms are described qualitatively, then the training phase is presented. The formal mathematical description of the model including all the equations is provided in the Supplementary Material, together with criteria for parameter assignments and parameter values.

### The neuro-computational model

#### Architecture

The model has been realized to simulate the behavioral tasks implemented by Crosse et al. (2022), which focuses on multisensory interactions in the time domain: subjects were required to respond to auditory and visual stimuli, alone or combined, presented in a random sequence, with an inter-stimulus-interval (ISI) varying between 1 s and 3 s. Given the experimental set-up of Crosse and colleagues, we do not require either multiple units sensitive to different spatial positions in each sensory area, as implemented in our previous neuro-computational models (Cuppini et al., 2014, 2017, 2018; Cuppini, Magosso, et al., 2011; Cuppini, Stein, et al., 2011; Ursino, Crisafulli, et al., 2017; Ursino, Cuppini, et al., 2017; Ursino et al., 2019), nor do we need multiple sensory regions sensitive to different input features, as necessary to realize semantic memory models (Ursino et al., 2010, 2011, 2018). Therefore, in the model, each region is simulated with a single neural element for simplicity.

**Fig. 1:**
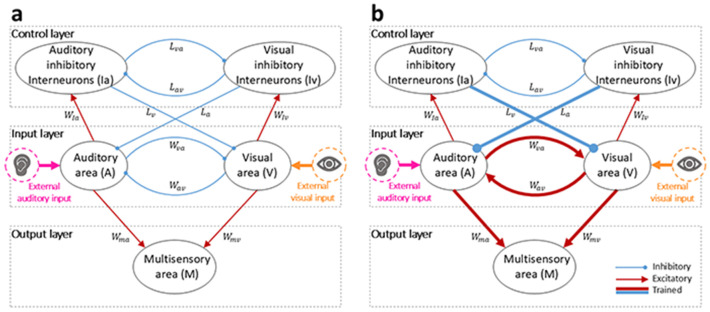
Model Structure. (**a**) Default configuration (simulating subjects of 6-9 years of age). A and V represent the auditory and visual regions, responsible for the first sensory processing, and implementing the input layer. They exchange direct inhibitory synapses, implementing a cross-modal competition. M is the multisensory output region. It is responsible for generating simulated reaction times (RTs) to the external stimuli. $I_{a}$ and $I_{v}$ are unimodal inhibitory areas, excited by the input layer and implementing a control mechanism via feedback inhibitory synapses and a winner-takes-all dynamics. (**b**) Adult configuration (simulating adults). Its synaptic architecture differs for i) the effectiveness of the excitatory feedforward $\left(W_{ma},W_{mv}\right)$ and inhibitory feedback $\left(L_{a},L_{v}\right)$ synapses; and ii) the now excitatory cross-modal synapses $\left(W_{av},W_{va}\right)$, implementing a cross-modal facilitation. Red lines represent excitatory connections; blue lines represent inhibitory synapses; bold lines were trained during the training phase.

The model structure consists of three layers (Fig. 1). An “input layer”, representing the primary unisensory auditory and visual areas (A and V in Fig. 1), receives external stimuli of the corresponding sensory modalities ($I_{0}^{a}$ and $I_{0}^{v}$, respectively) and provides the first sensory processing step. The onset, duration, and presentation rate (ISI) of the stimuli were chosen to mimic the experimental setup of Crosse et al., whose data are compared to our results. Specifically, external stimuli are excitatory inputs with an assigned efficacy, and a duration of 60 ms, presented at a mean rate of every 2 s (the mean ISI in Crosse et al.’s experiment).

The “control layer” ($I_{a}$ and $I_{v}$ in Fig. 1) implements cross-sensory competition between the two sensory modalities when stimuli are presented sequentially: modality-specific inhibitory interneurons produce a long-lasting inhibition of the input region processing external inputs of the other sensory modality. It is worth noting that, since the dynamic of this mechanism is slow, the processing of the current stimulus is affected by the previous one, when of the opposite sensory modality. This competitive layer can be implemented through higher order regions, for example located in the medial Prefrontal Cortex (mPFC) or the Posterior Cingulate Cortex (PCC). Huang et al. (2015) suggested that these regions may be involved in the competition between A and V sensory modalities, in a simple RT experiment. Hairston et al. (2008) reached a similar conclusion in the case of an auditory temporal order judgment (TOJ) task.

Finally, the third layer, a multisensory output area (M in Fig. 1), is used to mimic the behavioral responses (RTs) of subjects to external stimuli: the elicited activity in the output region is compared with a fixed threshold, φ (10% of the maximum neurons’ activity), to evaluate the simulated RTs, computed as the time interval between the instant of input presentation and the instant when the evoked activity in the output area reaches the threshold. This area corresponds to multisensory associative cortices, including, for example, the Posterior Parietal Cortex (PPC). Indeed, evidence from human intracranial recordings supports the role of the superior parietal lobule in audiovisual multisensory integration (Molholm et al., 2006).

#### Connectivity and implemented mechanisms

The two input regions send long range excitatory projections, $W_{ma}$ and $W_{mv}$, to the read-out multisensory layer (M region). Moreover, auditory and visual areas are reciprocally connected via cross-modal connections, $W_{av}$ and $W_{va}$. Both these synaptic mechanisms are characterized by fast dynamics. Various data in the literature confirm the existence of a direct influence between sensory cortices: anatomical studies in animal models have shown projections between core visual and auditory regions and associative areas (Cappe & Barone, 2005; Clavagnier et al., 2004), and revealed direct modulatory functional connections among sensory regions (Bizley & King, 2008; Meredith & Allman, 2015; Yu et al., 2013). Based on Monti et al. (2025), the default configuration of the model is characterized by inhibitory cross-modal synapses, mediating a multisensory competition mechanism: in the case of a multisensory stimulus, the input regions mutually suppress one another, resulting in a fast competition between the two sensory modalities. This competition balances the integration occurring in the M region (mediated by the excitatory feedforward connections projecting on it), nullifying any multisensory facilitation.

Additionally, neural elements belonging to the input layer send excitatory synapses ($W_{Ia}$ and $w_{Iv}$) toward the modality specific inhibitory areas ($I_{a}$ and $I_{v}$) of the control layer. This layer implements a winner-takes-all mechanism by means of reciprocal inhibitory synapses, $L_{av}$ and $L_{va}$, and the “winning” sensory modality exerts an inhibitory effect on the incoming stimulus of the opposite modality (if present), through feedback inhibitory synapses, $L_{a}$ and $L_{v}$. These synaptic connections are characterized by a slow dynamic, thus resulting in a long-lasting inhibitory effect.

The synaptic architecture of the adult configuration of the model (simulating adults) differs for i) the effectiveness of the excitatory feedforward ($W_{ma}$, $W_{mv}$) and inhibitory feedback ($L_{a}$, $L_{v}$) synapses; and ii) the now excitatory cross-modal synapses ($W_{av}$, $W_{va}$), implementing a cross-modal facilitation. The neural mechanisms implemented in the adult configuration are the same already used by Cuppini et al., 2020 to simulate the temporal profile of sensory processing in the adult human brain, in the cases of unisensory and multisensory stimulations.

It is worth noting that both models’ architectures incorporate two cross-modal mechanisms, characterized by different dynamics. The first cross-modal mechanism, mediated by the cross-modal connections between input areas, exhibits fast dynamics and, as a result, affects the current stimulus. This mechanism has an inhibitory nature in the default model configuration, and an excitatory nature in the adult configuration. The second cross-modal mechanism, on the other hand, is the one mediated by inhibitory feedback connections. Due to the slow dynamics of these synapses, this mechanism exerts its inhibitory effect on the processing of the subsequent stimulus (if it is of a different sensory modality than the preceding stimulus).

### Hebbian training

We sought to investigate the mechanisms behind the acquisition of integrative abilities, simulating the transition from the immature system, the so-called “default configuration”, to the adult condition. To do so, we repeatedly presented the model with modality-specific (A, V only) and congruent cross-modal stimuli (AV), and we strengthened synaptic connections according to Hebbian learning rules.

In particular, excitatory connections were modified by using a simple Long-Term Potentiation (LTP) Hebbian rule. Accordingly, correlated activation in the pre- and post- synaptic neurons leads to the strengthening of the connection between the two neurons (see supplementary materials for more details). The inhibitory synapses were reinforced using an anti-Hebbian rule: that is, a synaptic plasticity rule defined as the opposite of the classical Hebbian one, such that the connection is strengthened when the presynaptic neuron participates in the inhibition of the postsynaptic one (see supplementary materials for more details). Indeed, Hebbian learning is applicable only when the presynaptic neuron is excitatory. When the presynaptic neuron is inhibitory, Hebbian learning is contradictory: when pre- and post- synaptic neurons show a correlated activation, the strength of the connection (i.e., its weight or efficacy) should increase, thus resulting in a decreased efficiency in eliciting a response in the postsynaptic neuron. Therefore, for training these synapses, we implemented an anti-Hebbian rule.

To model sensory experience, we made a simplified choice, according to which only congruent cross-modal stimuli (AV), auditory stimuli alone (A-only) and visual stimuli alone (V-only) were used during the training phase; incongruent cross-modal audiovisual stimuli were not used for simplicity. The relative contribution of each of these stimuli during the maturational period was not available in the literature and presumably differs considerably across individuals and across the lifespan depending on circumstances. Therefore, we chose the stimulus configuration that best replicated the behavioral results in Crosse et al. (2022). Specifically, the network was stimulated with 30% AV stimuli, 50% V-only stimuli, and 20% A-only stimuli. Stimuli were generated through a uniform distribution of probability and presented in randomized order.

In order to compare our results with those of Crosse et al. (2022), we needed to relate the number of epochs during the training phase with the subject’s age. This choice, of course, depended on the values used for the learning rate (the higher the learning rate, the smaller the number of epochs). The parameters’ configuration used in the network’s default state, allowed to simulate RTs of 6–9 year-old subjects (the first data point present in Crosse et al., 2022). With the training parameters used, 500 epochs of training phase led to an architecture configuration yielding a behavior comparable to the 10-12 year-old subjects (the second data point present) in Crosse and colleagues’ data (Crosse et al., 2022). Therefore, in what is undoubtedly an oversimplification, 500 epochs of training were assumed to correspond to about 3 years of experience. According to this linear approach, 1000 epochs corresponded to exposures of 13-17 year-old subjects (the third data point present), and 1500 epochs should correspond to 18-20 years of age. Since the oldest age group represented in Crosse et al. (2022) is much larger than the other ones (it ranges from 18 to 40 years of age), the last data point has been obtained by mediating over 1500 training epochs (i.e., from 1500 to 3000). Overall, training involved 3000 exposures, at which point the network produced mature-like behavior.

Simulated RTs, during development, were analyzed separately based on the respective input combination, and compared with the empirical data of Crosse and colleagues.

### ASD simulations

Next, we sought to investigate what neural mechanisms differ in the autistic brain. In our previous work (Monti et al., 2025) we found that the parameter accounting for the difference between data from TD children and those from ASD children was the synaptic efficacy of cross-modal projections. Indeed, we previously found that these connections must be even more inhibitory to reproduce the RT data of ASD children.

Starting from the default configuration we previously identified for the autistic brain, we implemented and tested two different structural and functional hypotheses to simulate the delayed transition from the default state of competition (characterizing multisensory integration in childhood) to one of facilitation in ASD children. Specifically, we trained the network assuming either a reduced attention/exposure to audiovisual information (due to, for example, reduced fixations to the face due to face avoidance perhaps, intact fixation but reduced attention, both, and so forth) or a different level of synaptic plasticity. The reduced multisensory experience was simulated by presenting 20% of congruent cross-sensory auditory and visual stimuli, 40% of unisensory visual stimuli and 40% of unisensory auditory stimuli. We assumed that attentional biases are partly and progressively overcome by interventions and/or naturally occurring developmental changes. Indeed, recent theoretical (Beker et al., 2018) and computational (Cuppini et al., 2017) studies have proposed that constant exposure to cross-modal stimulation throughout development might serve as a training for multisensory functions, ultimately resulting in the gradual alleviation of multisensory deficits in individuals with ASD. In accordance with this notion, the number of multisensory events was progressively increased with age, and unisensory events were concurrently modified, to reach a TD-like multisensory experience in the final stage of development. To simulate the impact of reduced plasticity, we diminished the learning rates to 80% of those used in TD simulations. It is worth noting that, for these simulations, we only modified the learning rates, while the percentage of multisensory inputs was the same as for the simulations of TD children.

## Results

In the following section we present the results we obtained from our simulations. We have structured this section in a way that reflects its sequential nature, detailing each step we carried out, and elucidating how each step influenced the subsequent one. Overall, these results allowed us to understand (i) what plastic neural changes occur during the maturational period and (ii) what differs in ASD development. Finally, we discuss a possible intervention, based on multisensory stimulation, to fasten the recovery of perceptual and integrative abilities in ASD.

### The default configuration of the model reproduces and explains children’s empirical data

The results of all computational simulations were compared with the empirical data collected by Crosse and colleagues (Crosse et al., 2022). As far as we know, this is the only study in the literature describing the maturation of perceptual abilities in the temporal domain, and the task-switch cost, in children with typical development (TD) and that with an autism spectrum diagnosis (ASD). As such, to test the implemented mechanisms, we simulated the same results described in that experimental effort, considering the same age-groups: children between 6-9, 10-12, 13-17 and >17 years of age. It is worth noting that in Monti et al. (2025), we simulated the behavior of the youngest children (i.e., 6 years of age), not of the youngest age group (i.e., 6-9 years of age). Therefore, we initially set the model to the default configuration described in Monti et al. (2025), which was characterized by inhibitory cross-modal interactions and capable of explaining the behavioral data of 6-year-old children. We then adjusted the parameters values to reproduce the entire 6-9 year-old age group.

In the following, to visually demonstrate how the model works in its initial configuration, we examine the network’s responses under different circumstances with the parameters we found (i.e., comparing unisensory against multisensory stimulation and repeat against switch trials). The simulated behavior is compared with the empirical RT data.

**Fig. 2:**
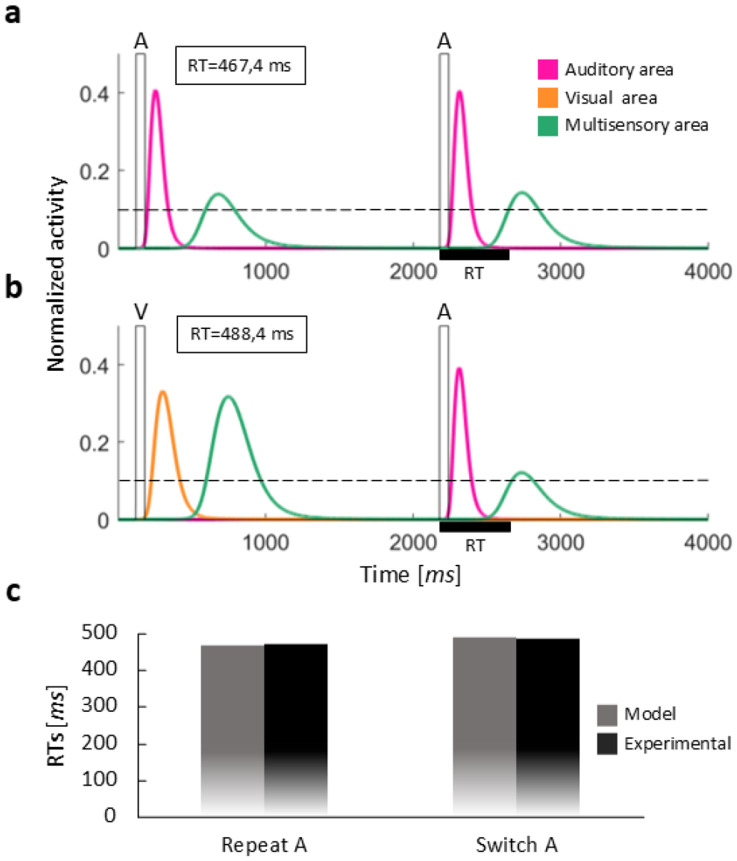
Network behavior in the auditory conditions: auditory repeat vs auditory switch. (**a**) Example of the network behavior in the auditory repeat condition (A→A). The two rectangular impulses represent the external auditory stimuli presented with an ISI of 2000 ms. Light and dark grey lines describe the activation of auditory and visual regions, respectively. Black lines represent the activity elicited in the output region in response to the stimulation. This activity is compared with the detection threshold (dashed line): the simulated RT (horizontal black bar) is calculated as the interval between the instant when the black line overcomes the threshold and the instant of stimulus presentation. (**b**) Example of the network behavior in the auditory switch condition (V→A). The two rectangular impulses represent the external auditory and visual stimuli presented with an ISI of 2000 ms. Conventions are the same adopted in the previous panel. In the repeat condition, the RT is slightly faster than the RT in unisensory switch condition. (**c**) Comparison between the simulated RTs obtained with our model and the median RTs, extracted from the real population (Crosse et al., 2022), for auditory repeat and switch conditions.

**Fig. 3:**
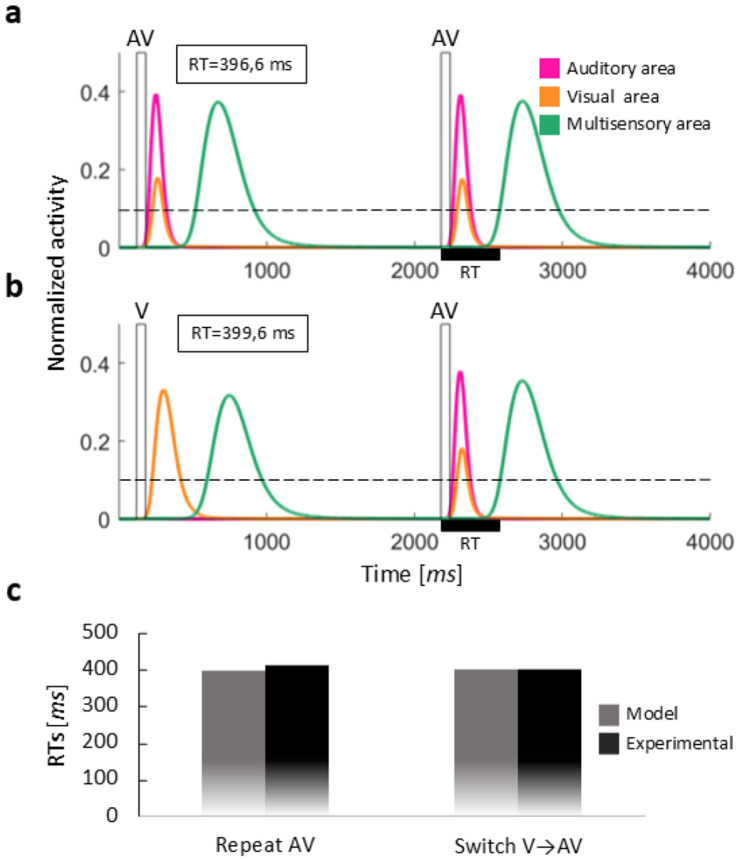
Network behavior in the multisensory conditions: audiovisual repeat vs audiovisual switch. (**a**) Example of the network behavior in the audiovisual repeat condition (AV→AV). The two rectangular impulses represent the external audiovisual stimuli presented with an ISI of 2000 ms. Conventions are the same adopted in the previous figure. (**b**) Example of the network behavior in an audiovisual switch condition (V→AV). The two rectangular impulses represent the external visual and audiovisual stimuli presented with an ISI of 2000 ms. Conventions are the same adopted in the previous figure. In the multisensory switch condition, the RT is comparable to the RT in repeat condition. (**c**) Comparison between the simulated RTs obtained with our model and the median RTs, extracted from the real population (Crosse et al., 2022), for audiovisual repeat and switch conditions.

Fig. 2 and Fig. 3 compare the activities evoked in the regions of the model in case of auditory repeat (A → A) versus auditory switch (V → A), and audiovisual repeat (AV → AV) versus audiovisual switch (V → AV), respectively. These figures serve to illustrate how the RTs are computed in the different conditions. It is worth noting that auditory and visual stimuli always have the same efficacy (black rectangles in the figures) and the second stimulus is presented with an ISI of 2 s in all the trials. Therefore, stimulus efficacy and ISI are not responsible for the different activities evoked in the regions of the model, which depend only on the structure of the network and the specific stimulus configurations.

Comparing auditory repeat and switch conditions (i.e., switch vs repeat for unisensory trials, Fig. 2), we noted that the activity elicited by the second stimulus in the auditory region of the model (pink lines in the figures) is slightly lower in the switch case. This reduced activation of the A region in the switch condition is due to the inhibitory interneuron excited by the preceding visual stimulus: the visual interneuron sends to the input region of the opposite sensory modality (i.e., the A region) an inhibitory contribution characterized by a slow dynamic (i.e., the effect acts over a long time). The resulting lower activation of the auditory region produces a delayed activation of the multisensory region, with a consequently longer RT, that is the so-called switch cost. It is worth noting that the activity elicited in the auditory region by the second stimulus is only slightly lower in the switch condition compared to the repeat condition; similarly, the RT is only slightly slower. This is caused by the low efficacy of the inhibitory feedback synapses (driving the inhibitory input), that are still immature in the default configuration.

Comparable results were found in the case of visual repeat/switch trials (results not shown for briefness): in the switch condition, the preceding auditory stimulus lowers the activity evoked in the visual input region, thus resulting in a slower activation of the M area and a slower response. As described for the auditory switch/repeat conditions, this effect is produced by the auditory-driven long-lasting inhibition to the visual input region.

Comparing the activities and the RTs in the cases of auditory and audiovisual repeats (i.e., unisensory vs multisensory stimulation, Fig. 2A and Fig. 3A), we observed that, in case of multisensory repeat, the activity elicited in the M area (green lines in the figures) is higher than the activity in the auditory repeat condition, for both the first and the second stimulus. This higher activation of the multisensory region is due to the feedforward connections converging upon it: these projections implement a multisensory integration effect that produces stronger activity in the output region, and therefore quicker RTs in response to AV stimulation, compared to the unisensory conditions.

Additionally, according to Crosse et al.’s data (Crosse et al., 2022), contrary to unisensory trials, multisensory trials are not characterized by a switch cost, indeed multisensory repeat and multisensory switch RTs did not differ significantly. Our simulations show a similar pattern of results (Fig. 3A and Fig. 3B). The absence of a switch cost in multisensory trials can be explained by the presence of long-lasting cross-modal inhibition. In the switch condition, the preceding unisensory stimulus inhibits the unisensory component of the following AV stimulus that belongs to the opposite modality. In the repeat condition, both unisensory components of the preceding AV stimulus could, in principle, exert inhibition on the unisensory components of the following AV stimulus. However, a winner-takes-all mechanism between inhibitory interneurons ensures that only one of them is active and sends inhibition to the opposite unisensory input region. As a result, the net inhibitory effect is similar in both repeat and switch multisensory trials, leading to comparable RTs. All other mechanisms involved are the same as those already described for the multisensory repeat condition.

Fig. 2C and Fig. 3C compare the simulated RTs with empirical RTs of the corresponding conditions, showing that the model in its default configuration provides a good simulation of the empirical data from 6-9 year-old children in Crosse et al. (2022).

### Development of sensory interactions for TD

We next sought to reveal the mechanisms underlying the age-related changes observed by Crosse and colleagues (Crosse et al., 2022). Crosse and colleagues (Crosse et al., 2022) revealed that the interactions between auditory and visual processes, in simple perceptual tasks, shift from competitive to facilitatory during late childhood and early adolescence. To model how this happens in the brain and the modifications occurring in the implemented neural mechanisms, in a second set of simulations we trained the model with an Hebbian learning paradigm, and we analyzed the modifications of the network’s architecture resulting from the training procedure, comparing these changes with the developmental trajectories of Crosse’s subjects. To simulate and match the analysis performed on the empirical data, subdivided into four age-groups (i.e., 6-9 years of age, 10-12, 13-17, ≥18), the network’s performance was tested at various epochs of maturation, assuming that 500 training epochs corresponded to 3 years span.

First, we assessed which synaptic connections to train. Feedforward connections must be trained because Crosse and colleagues (2022) observed an age-related effect, with older participants responding faster than younger participants. Similarly in the model, as feedforward connections strengthen over the training epochs, the M area overcomes the detection threshold earlier, thus resulting in faster RTs. Feedback inhibition was trained as well, because Crosse et al. (2022) revealed that the so-called switch cost, that is the longer RTs when the sensory modality switches (A → V, V → A, A → AV, V → AV) compared to when it repeats (A → A, V → V, AV → AV), increases during development. We reasoned that the switch cost would remain constant if feedforward connections were strengthened alone. This suggests that feedforward synapses and feedback inhibition must evolve in a balanced fashion in order to accurately reproduce experimental RT data. In fact, as excitatory connections to the M area strengthen, RTs become faster for all stimulus configurations. However, the parallel reinforcement of inhibitory feedback reduces the activity of unisensory areas in switch conditions, balancing the reduction of RTs produced by the development of feedforward connections and resulting in progressively longer switch costs. Finally, the reciprocal cross-modal projections were trained to shift from the initial competitive cross-sensory organization to one of facilitation, as observed from developmental empirical data where RTs to cross-modal stimulations (AV Switch and Repeat conditions) in children are not faster than unisensory conditions, while in the adult populations, AV RTs are faster than any other stimulus configuration (Crosse et al., 2022).

**Fig. 4:**
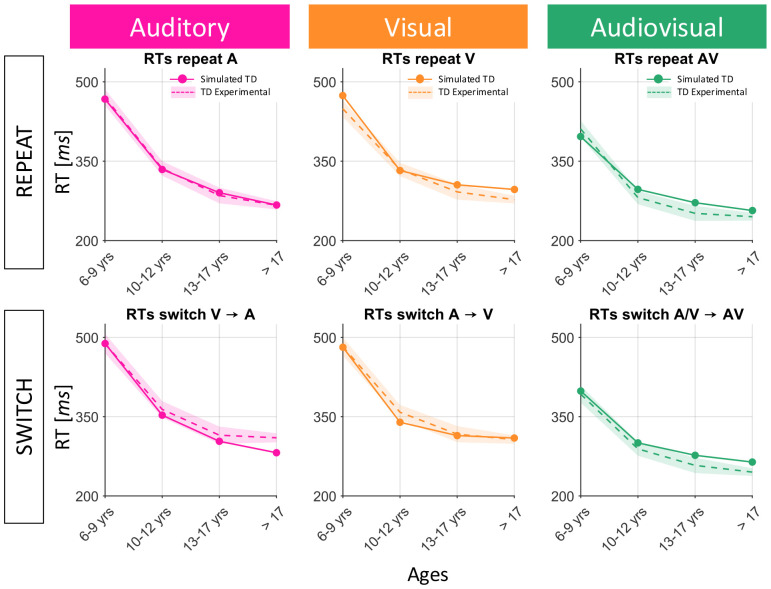
Comparison between model training results and empirical developmental trajectories of TD subjects. Simulated RTs, in the repeat (upper panels) and switch (lower panels) conditions, for each sensory modality (RTs to A stimulation in the left panels; RTs to V stimulation in the central panels; RTs to AV stimulation in the right panels), are evaluated at different training epochs (solid lines) and compared with experimental data of TD children (Crosse et al., 2022) of different ages (dashed-dotted lines).

Fig. 4 shows the development of RT performance for TD children. The output of the network (solid lines) is compared with the empirical data described by Crosse et al. (2022) (dashed-dotted lines) at different phases of training, simulating years of age (from childhood, through adolescence, to an adult condition), for all six experimental conditions: auditory repeat and switch (blue lines in figures), visual repeat and switch (purple lines in figures) and multisensory repeat and switch (orange lines in figures).

Unisensory and multisensory perceptual capabilities develop with a similar maturational pattern, for both repeat and switch conditions. In both cases, the network reached “adult-like” levels after 1500 training epochs (corresponding approximately to 17 years of age), at which point cross-modal connections have become excitatory, while feedforward projections toward the M area and the inhibitory feedback synapses have strengthened (boosting their excitatory and inhibitory effects, respectively).

After this training, the mature architecture of the model implements two multisensory effects, one facilitatory and the other inhibitory.

- The input layer and the multisensory area, with their specific synaptic architecture (i.e., the reciprocal excitatory connections, and the converging feedforward synapses), characterized by fast dynamics, provide the neural substrates for the multisensory processes performed in response to an external input, and, in the case of audiovisual stimulation, implement multisensory facilitation.
- The feedback projections to the input layer from the competitive layer, described by slower dynamics, realize competition between the sensory modalities.

Importantly, whereas in the default configuration, auditory and visual areas interact in a competitive fashion, mutually suppressing each other, in the final configuration (i.e., simulating adults), after the training, input regions are noncompetitive, being interconnected through excitatory cross-modal synapses. In other terms, while in the naïve state, multisensory responses were dictated by a principle of competition, when multimodal cues are experienced over many training iterations of the model, the cross-modal pathway is strengthened and becomes excitatory. Thereafter, multisensory responses are driven by cooperative cross-modal interactions. Overall, this is in accordance with the results of Crosse et al. (2022) and previous studies (Yu et al., 2019).

The agreement between the model’s behavior and the empirical data is good, as expected, since the parameters of the model were set to reproduce these data. To assess quantitatively the goodness of fit between the model and the empirical data, we performed a 1-way ANOVA with factor of data source (model against empirical data). This analysis showed no main effect of data source (*F*(1,46) = 0.02, p = 0.8883).

### Testing the effect of different “perturbations” to unveil developmental processes in ASD

In a subsequent series of simulations, we aimed to elucidate the various mechanisms contributing to the delayed development of perceptual abilities in children with ASD. First, as we did for TD children, we explored the optimal set of parameters for reproducing the behavior of children aged 6-9 years.

Building upon our prior research (Monti et al., 2025), cross-modal connections must be more inhibitory compared to those in TD children in order to accurately replicate RT data in children with ASD (Table S1).

**Fig. 5:**
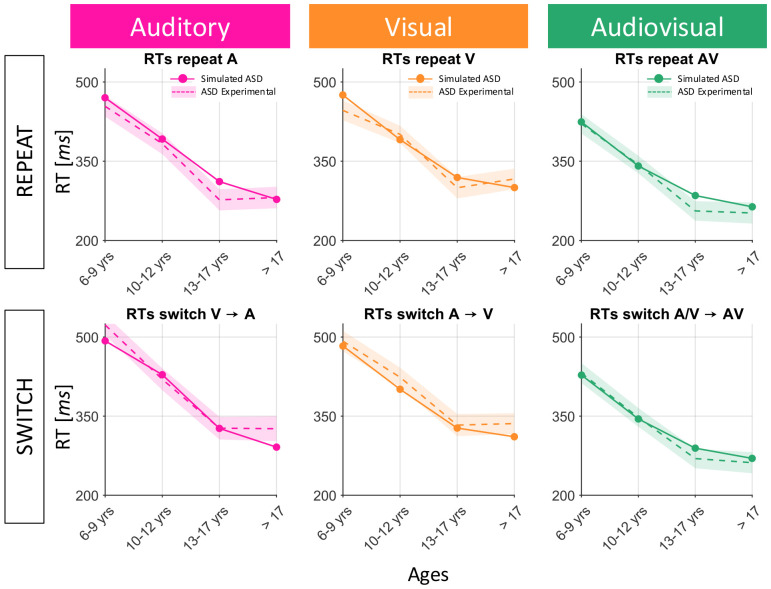
Comparison between empirical developmental trajectories of ASD subjects and model training results with reduced plasticity. Conventions are the same adopted in the previous figure. Simulated RTs (solid lines), obtained perturbing the network with a reduced plasticity, are evaluated at different training epochs, and compared with experimental data of ASD children (Crosse et al., 2022) of different ages (dashed lines).

**Fig. 6:**
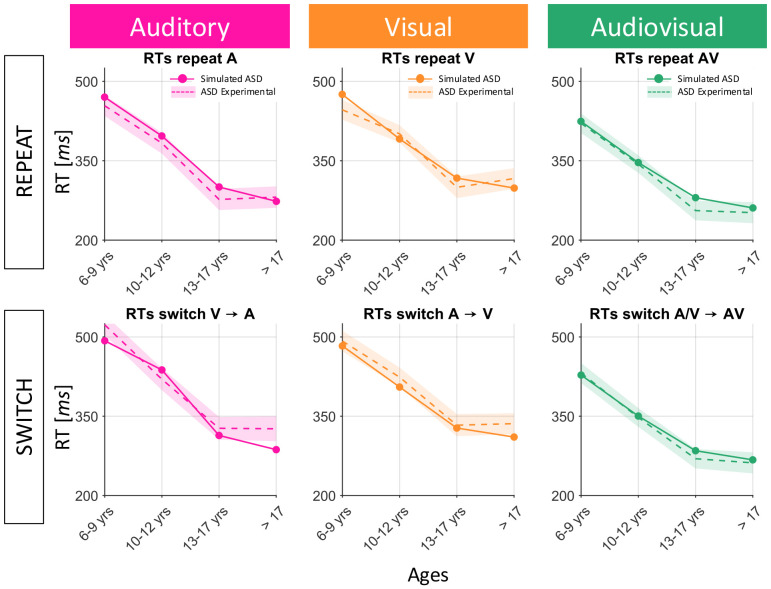
Comparison between empirical developmental trajectories of ASD subjects and model training results with reduced multisensory AV exposure. Conventions are the same adopted in previous figures. Simulated RTs (solid lines), obtained perturbing the network with a reduced percentage of AV stimuli presentation, are evaluated at different training epochs, and compared with experimental data of ASD children (Crosse et al., 2022) of different ages (dashed lines).

After setting the initial neural architecture, reduced multisensory exposure and reduced plasticity were tested as possible explanations of the delayed maturational trajectories of ASD children. Numerous studies in the literature have suggested that both of these factors may be dysregulated in individuals with ASD (please refer to the discussion section for additional details). In the following paragraph, we analyze both training hypotheses and the corresponding results, to clarify how they could explain the behavioral data of ASD subjects, and to identify if one of the two hypotheses better reproduces the changes in ASD subjects’ RTs across development. To this aim, the output of the network (solid lines in Fig. 5 and Fig. 6), at each sampled training epoch and for all six experimental conditions, was compared with the empirical developmental data of ASD children (dashed lines in Fig. 5 and Fig. 6).

#### Reduced synaptic plasticity

Recurrent findings in the study of Autism are dysregulated experience-dependent long-term synaptic depression (LTD) (for review, see Hansel, 2019) and abnormalities in the expression of synaptic receptors (Aguilar-Valles et al., 2015; Hadley et al., 2014). Consequently, ASD is typically associated with altered synaptic transmission and plasticity. These observations led us to hypothesize that reduced plasticity may play a crucial role in slowing down ASD individuals’ perceptual abilities, and we tested this hypothesis by reducing the learning rates. Our results (Fig. 5) indicate that reduced plasticity affects the maturation of unisensory and multisensory perceptual abilities in a similar way: in both cases the developmental trajectories obtained with the model are delayed compared to those obtained for simulated TD children (i.e., with a normal plasticity level). This effect is evident in the first two training steps (i.e., between 6-9 years of age and 10-12 years of age, and between 10-12 years of age and 13-17 years of age). Perceptual capabilities (either unisensory or multisensory) became comparable to the TD condition in the last training step (i.e., between 13-17 age group and the adult group). These results suggest a delayed maturation occurring between 6 and 17 years of age in case of reduced plasticity, whilst perceptual deficits are not evident anymore at later training phases. Overall, this is in agreement with the consolidated notion that perceptual abilities in ASDs develop more slowly than in TDs, but then catch up with “TD-like” behavior during late adolescence or early adulthood. In general, network simulations well reproduced the empirical data. Supporting the good fit between model simulations and experimental RTs, a 1-way ANOVA with factor of data source (model against empirical data) was performed. This statistical analysis showed no main effect of data source (*F*(1,47) = 1.24, p = 0.2707). As such, reduced plasticity is a possible explanation accounting for the pattern of perceptual deficits observed in ASD.

#### Reduced multisensory exposure

Given that early multisensory experience is fundamental for the development of integrative abilities (Bahrick & Lickliter, 2000; Carriere et al., 2007; Rowland et al., 2014; Santapuram et al., 2022; Wallace, 2004; Wallace et al., 2004; Wallace & Stein, 1997, 2007; Xu et al., 2014; Yu et al., 2010), it is conceivable that the impaired integrative and perceptual abilities of ASD children and their delayed maturation could be due to experiencing fewer multisensory stimuli (Santapuram et al., 2022). To test this possibility, we reduced the percentage of multisensory stimuli presented to the network. Specifically, we trained the network starting with 20% of AV stimuli, 40% A and 40% V; then we increased the multisensory experience by 10% every 500 epochs (simulating about 3 years), changing consequently also the percentage of A and V stimuli. As is evident from Fig. 6, the effects of this perturbation on the simulated developmental trajectories are similar to those described in the case of reduced plasticity. That is, the maturation is delayed, compared to the simulated TD development, until reaching the third data point, simulating the 13-17 year-old group. Therefore, similar to the case of reduced plasticity, the hypothesis of a reduced multisensory exposure also predicts that development is delayed, compared to the TD one, before 17 years of age, but then ASD perceptual and integrative abilities catch up with TD-like behavior.

Also in this case, network simulations provide a good reproduction of the empirical data. We performed a 1-way ANOVA with factor of data source (model against empirical data), to test the good fit between model simulations and experimental RTs. This statistical analysis showed no main effect of data source (*F*(1,47) = 1.16, p = 0.2876). As such, reduced multisensory experience provides a good account of the pattern of deficits seen in ASD.

### Multisensory interventions reduce developmental delay in the ASD model

Higher-order cognitive functions hierarchically depend on early perceptual processing stages. Therefore, given the previous observations and considering the cascade effect that perceptual dysfunctions may have on core ASD symptoms and the importance of clinical implications when dealing with clinical populations, we decided to further exploit our model for testing the effects of a possible multisensory rehabilitative strategy.

We found that both perturbations introduced to simulate ASD development—reduced synaptic plasticity and limited multisensory experience—decreased the effectiveness of the Hebbian learning rule. If we aim at identifying and testing a possible intervention to speed up the acquisition of perceptual abilities in this population, we need to identify a method to improve the efficacy of the Hebbian rule, in the simulated maturation. Since plasticity cannot be modulated, the only way to obtain this result is by increasing the exposure to AV stimuli. That is, forcing a sort of intensive multisensory training. To test this hypothesis, we trained the network using a high percentage of AV stimuli (60%), either in the case of reduced plasticity or in the case of reduced multisensory exposure.

#### Benefit of multisensory interventions on reduced synaptic plasticity

For these simulations we used the same learning rate values used for testing the impact of reduced plasticity on the network behavior. No other parameters changed compared to the default configuration used for simulating the behavioral data of ASD children of 6-9 years of age, other than the relative percentages of stimuli presented. Indeed, multisensory exposure was forced to be extremely high (much higher also compared to the sensory experience of TD children). In particular, we used 60% AV inputs, 10% A stimuli and 30% V stimuli for the entire duration of the training phase.

**Fig. 7:**
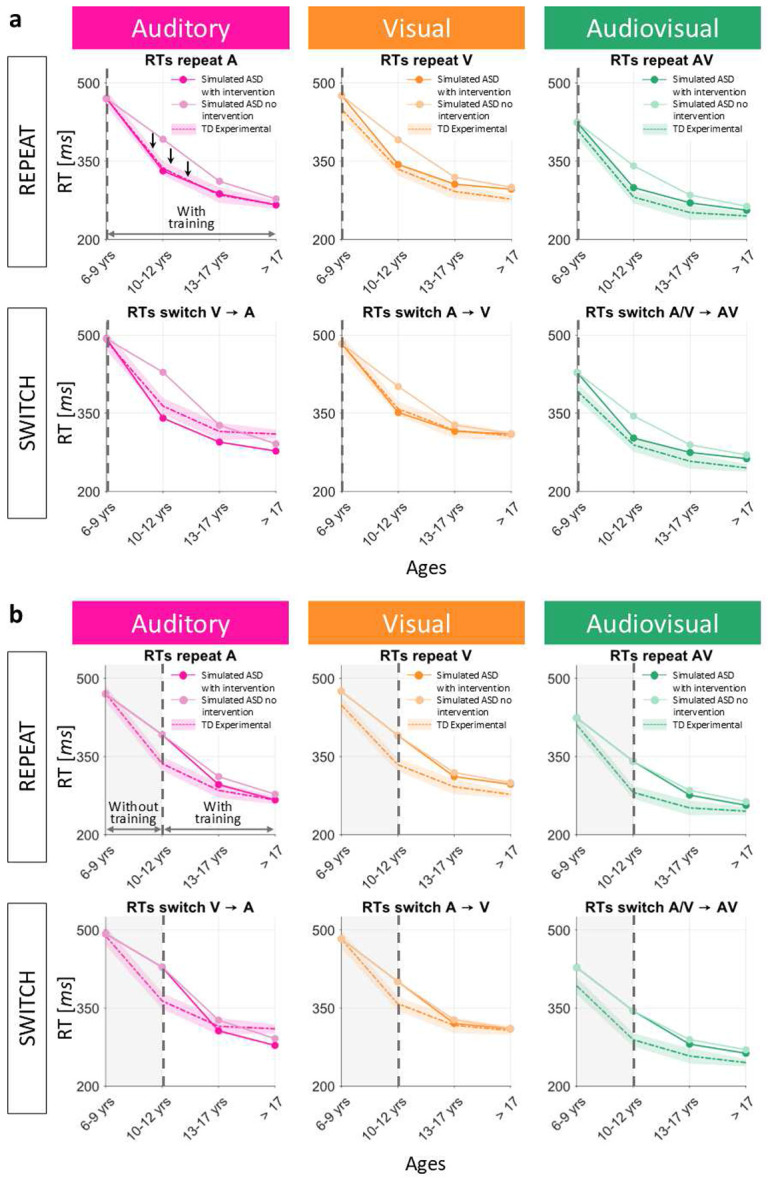
Effects of the highly multisensory intervention in case of reduced plasticity. (**a**) The multisensory-biased training, implemented by training the network presenting 60% of AV inputs, is applied starting from the very beginning of the training phase. The so obtained simulated RTs are sampled at different stages of the training and compared with simulated RTs obtained in case of reduced plasticity, and with the empirical RTs of TD children of the corresponding age group. The maturational delay is quickly recovered and the simulated RTs become comparable with the empirical TD ones. (**b**) The same highly multisensory training is applied starting from the second age group only. The so obtained simulated RTs are sampled at different stages of the training and compared with simulated RTs obtained in case of reduced plasticity, and with the empirical RTs of TD children of the corresponding age group. An improvement is still present, but it is very limited.

The benefits of the intervention were evaluated by comparing the obtained maturational trajectories with those obtained for the simulated ASD subjects under the hypothesis of reduced plasticity and with the empirical developmental data of TD children. The results (Fig. 7A) show that when the proposed intervention is applied immediately (i.e., starting from the very beginning of the training, at 6 years of age), the simulated RTs move away from the developmental trajectories obtained under the hypothesis of reduced plasticity and normal AV experience, and come closer to the TD experimental maturational curves. ANOVA analysis revealed no significant differences between simulated ASD developmental trajectories with intervention and empirical TD development (*F*_1/47_ = 0.06, *p* = 0.8053), and between simulated ASD developmental trajectories with intervention and the development simulated for ASD without intervention (*F*_1/47_ = 0.85, *p* = 0.3619). Nevertheless, this analysis quantitatively showed that ASD developmental trajectories with the simulated intervention are closer to the empirical TD developmental pattern than to the simulated ASD development without intervention.

We also tested the effects of the same intervention when applied later, starting from the second age group (i.e., 10-12 years of age). In this case, during the first training step (i.e., from 6-9 years of age to 10-12 years of age), all the parameters were identical to those used to simulate the ASD with a training characterized by a reduced plasticity. Then, from the second training step on, the percentage of AV stimuli was increased to 60%, and maintained fixed until the end of the training. Our results (Fig. 7B) predict that, if the intervention is applied later, an improvement in terms of faster RTs is still present, but it is very limited compared to a timelier intervention. Also in this case, ANOVA did not show any significant difference between conditions, but revealed that, if the same intervention is applied later, simulated ASDs’ trajectories with intervention are quantitatively closer to the simulated ASDs’ trajectories without intervention (*F*_1/47_ = 0.04, *p* = 0.8355), than to the experimental TD developmental pattern (*F*_1/47_ = 0.89, *p* = 0.3512).

#### Benefit of multisensory interventions on reduced multisensory exposure

**Fig. 8:**
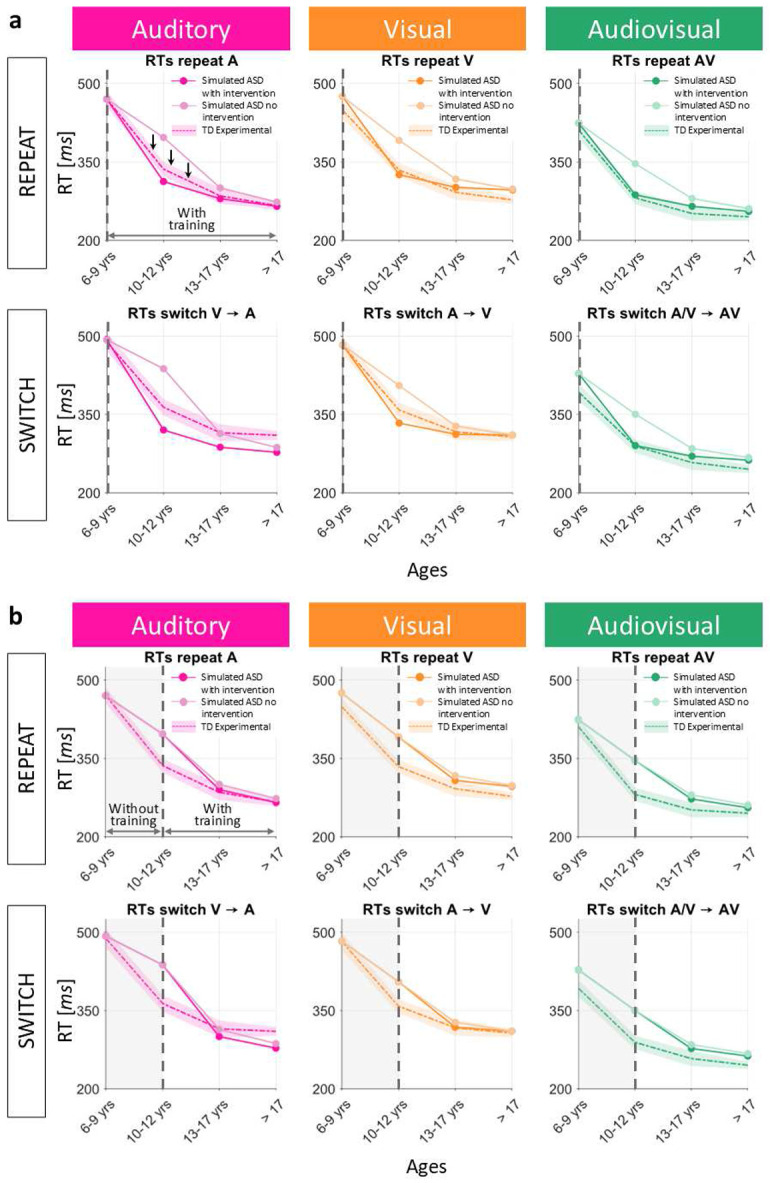
Effects of the highly multisensory intervention in case of reduced multisensory experience. (**a**) The multisensory-biased training, implemented by training the network presenting 60% of AV inputs, is applied starting from the very beginning of the training phase. The so obtained simulated RTs are sampled at different stages of the training and compared with simulated RTs obtained in case of reduced AV exposure, and with the empirical RTs of TD children of the corresponding age group. The maturational delay is quickly recovered and the simulated RTs become comparable with the empirical TD ones. (**b**) The same highly multisensory training is applied starting from the second age group only. The so obtained simulated RTs are sampled at different stages of the training and compared with simulated RTs obtained in case of reduced AV exposure, and with the empirical RTs of TD children of the corresponding age group. An improvement is still present, but it is very limited.

The same intervention was tested in the case of reduced multisensory experience as the cause of the delayed maturation in ASD. For these simulations, all the parameter values were the same as those used for simulating the ASD behavioral data, with the only exception being the percentage of AV stimuli, which was forced to be extremely high. Also in this case, the network was presented with 60% AV inputs (i.e., multisensory exposure much higher than that used for simulating TD child development), 10% A stimuli and 30% V stimuli, for the entire training phase. Comparing the so obtained simulated developmental trajectories with those obtained for the simulated ASD children under the hypothesis of reduced multisensory experience, and with the empirical developmental curves of TD children (Fig. 8A), we could observe that, when the intervention is promptly applied, the simulated RTs improve quickly, becoming “TD-like” already in the first training phases. Additionally, although statistical comparisons are not significant, under the hypothesis of reduced multisensory exposure, ASD developmental trajectories with the simulated intervention are closer to the empirical TD development (*F*_1/47_ = 6.2822· 10^−6^, *p* = 0.998) than to the simulated ASD development without intervention (*F*_1/47_ = 1.22, *p* = 0.2756).

For completeness, we tested the effects of the same intervention, applied later, under the hypothesis of reduced AV exposure: during the first training step (i.e., from 6-9 years of age to 10-12 years of age), all the parameters were identical to those used when training the network with reduced multisensory experience and simulating ASD children. Then, from the second training step on, the percentage of AV stimuli was increased (60%) and maintained at this fixed level until the end of the training. Also in this case, if this more intense multisensory training is applied later, the effects are not so evident as when it is applied immediately (Fig. 8B). Similar to the results obtained under the hypothesis of reduced plasticity, in the case of reduced multisensory exposure, if the intervention is applied later, simulated ASD trajectories with intervention are closer to the simulated ASD trajectories without intervention (*F*_1/47_ = 0.03, *p* = 0.8739) than to the experimental TD developmental pattern (*F*_1/47_ = 0.88, *p* = 0.3541). However, these comparisons did not reach statical significance.

## Discussion

Our results suggest that the transition from a state of competition between sensory modalities to one characterized by mutual facilitation (Crosse et al., 2022; Medina et al., 2023; Monti et al., 2025; Yu et al., 2019) is mediated by the strengthening of direct cross-modal synapses. These connections, inhibitory at birth, develop based on our experience with cross-modal stimuli, and transition to an becoming excitatory state across the course of development. The idea that primary sensory cortices do not work in isolation, but are substantially influenced by other sensory modalities is firmly established in the literature (Driver & Noesselt, 2008; Foxe et al., 2000; Foxe & Schroeder, 2005; Ghazanfar & Schroeder, 2006; Molholm et al., 2002; Musacchia & Schroeder, 2009; Recanzone, 2009; Schroeder & Foxe, 2005; Shams & Kim, 2010; Stein & Stanford, 2008). The fact that these connections are shaped by multisensory experience should not come as a surprise. Indeed, the repeated and simultaneous (or within a limited time window) activation of multiple unisensory areas, resulting from exposure to multisensory cues, leads to an increase in the synaptic efficacy of cross-sensory connections (Hebb, 1949). Our work builds upon the hypothesis recently proposed by Yu et al. (2019), which suggests that the default state of cross-modal interactions (at birth) is one of competition. This hypothesis challenges the classical view, which posits that initially, cross-modal interactions are in a state of “non-interaction” among the senses (Piaget, 1952). Multisensory experience then allows for the emergence of the classic cross-modal facilitation observed in adults. Yu and colleagues' study was based on an animal model of multisensory integration, specifically the authors recorded the activity of single neurons in the superior colliculus of cats (one of the most studied integrative hubs to understand the rules subserving multisensory integration). However, recent computational (Monti et al., 2025) and behavioral (Crosse et al., 2022) work has suggested that something similar also occurs in humans. Here, we provide further insights into this aspect, presenting plausible neural mechanisms responsible for the transition from an initial state of cross-modal competition to the mature state of cooperation. We suggest that this transition is mediated by the evolution of cross-modal connections, which also undergo a shift from inhibitory to excitatory states.

Another primary objective of this study was to offer an explanation for the delayed development of cross-modal interactions in children with ASD. Our model provides evidence that reduced exposure to multisensory stimuli and/or diminished synaptic plasticity might slow down the maturation of cross-modal connections, leading to a delayed transition from cross-modal competition to facilitation. This idea is supported by previous research, which has often found abnormalities in synaptic plasticity markers, mechanisms, and receptors, in individuals with ASD (Aguilar-Valles et al., 2015; Guang et al., 2018; Hadley et al., 2014; Hansel, 2019; Lee et al., 2022; Nisar et al., 2022; Zoghbi & Bear, 2012), resulting in differences in LTD, critical for proper developmental synaptic pruning and brain functional development. Early exposure to multisensory cues plays a crucial role in the development of integrative skills (Bahrick & Lickliter, 2000; Carriere et al., 2007; Ross et al., 2011; Rowland et al., 2014; Wallace, 2004; Wallace et al., 2004; Wallace & Stein, 1997, 2007; Xu et al., 2014; Yu et al., 2010); thus, reduced experience with multisensory stimuli in individuals with ASD might contribute to impaired integrative and perceptual abilities and delayed maturation. This hypothesis aligns with the Weak Central Coherence Theory, one of the earliest and foremost theories of Autism (Frith, 1989; Happé & Frith, 2006). According to this theory, individuals with autism have a sensory processing style characterized by a reduced ability to integrate and process information across different perceptual and cognitive domains, leading to a preference for processing information in a more fragmented or detail-oriented manner. Because combining information from multiple senses to form a coherent perception of the world can be challenging for children with ASD, their experience with multisensory stimuli is disrupted, hindering the development of multisensory circuits and functions.

However, it is important to note that multisensory deficits in ASD children may not be solely attributed to reduced multisensory experience or altered synaptic plasticity. For instance, it is entirely plausible that a combination of both reduced experience and altered plasticity contributes to these deficits, and other factors may well also play a role. Overall, more research is needed to better understand the specific mechanisms involved and their relative contribution to multisensory deficits in Autism.

The purpose of the present study was to replicate and explain the RT data presented by Crosse et al. (2022), clarifying the neural mechanisms at play during a bisensory reaction time task at various stages of development. While the maturation of cross-modal projections accounts for the developmental trajectories of cross-modal interactions, in both TD and ASD children, the maturation of other synaptic connections explains age-related changes in RTs and Switch Cost observed by Crosse and colleagues. On one hand, our simulations accurately predict the developmental pattern of RTs, reproducing their decrease with age. The model explains this developmental pattern with the strengthening of feedforward connections, projecting from lower-order to higher-order cortices. On the other hand, Crosse and colleagues observed a slight but constant increase in Switch Cost during development (Fig. 6A in Crosse et al., 2022), which our model attributes to the combined maturation of both feedforward synapses and inhibitory feedback synapses. Indeed, our model indicates that training the feedforward connections alone was not sufficient, as it resulted in a negligible increase in the Switch Cost. Additionally, according to our empirical data, the Switch Cost does not change significantly across development in the multisensory condition. The model also accounts for this fact: the development of inhibitory feedback, which is the one underlying the increase in the MSE in unisensory trials, is compensated for by the maturation of excitatory cross-modal connections in the multisensory trials. Overall, our model has unveiled a complex interplay between cross-modal interactions and sensory modality-switching. Their relative contribution in determining the RTs evolves dynamically during development.

These findings have important implications for the understanding of neural mechanisms underlying higher-order cognitive dysfunctions in ASD individuals. Indeed, any high-level cognitive process relies on the entire chain of preceding neural processing stages, tracing back to the very earliest stages of sensory processing. In other words, our behavior ultimately depends on how we perceive the external world. In this view, multisensory and perceptual deficits of ASD children could negatively impact higher cognitive functions, including social interactions and communicative functions (Foss-Feig et al., 2012; Robertson & Baron-Cohen, 2017; Ross et al., 2015, 2024; Siemann et al., 2020; Stevenson, Segers, et al., 2014), which are the core symptoms associated to autism (American Psychiatric Association, 2022). This opens up exciting new opportunities for linking various levels of brain processing, which is essential for the development of novel rehabilitation approaches. Indeed, by developing simple perceptual training tasks, it may well be possible to target complex cognitive functions as well, acting through the entire chain of cascading neural processes. We argue that such an intervention strategy is not only easier to implement, but also much more robust (e.g., Schaaf et al., 2025).

This potential clinical translation prompted us to utilize the model as a translational neuroscience framework for predicting the effect of rehabilitation strategies. Both the perturbations we tested as plausible explanations for the delayed development of MSI in ASD resulted in a reduced efficacy of the Hebbian rule (i.e., there is a learning deficit). We reasoned that it would be possible to increase again the efficacy of the training rule by presenting the network with a very high amount of AV stimulation. This can be conceptualized as providing ASD children with intensive multisensory training. Simulations suggested that an intervention strategy focused on multisensory stimulation could accelerate the amelioration of multisensory deficits in ASD children, and, possibly, also core higher-level symptoms (e.g., social communication and interactions). In particular, the model predicts different training outcomes based on the timing of the training intervention: perhaps unsurprisingly, the earlier this intervention is applied, the more beneficial it is, and the faster ASD integrative abilities reach TD-like levels. This result is consistent with previous studies indicating that, even if a considerable amount of brain changes, including synaptic remodeling, occurs well beyond adolescence and stabilizes in adulthood (Petanjek et al., 2011), there are some critical periods at which the brain development is particularly sensitive to experience (Hensch, 2005).

Finally, it is worth noting that our model provides a mechanistic explanation for the Weak Central Coherence Theory (Frith, 1989; Happé & Frith, 2006), showing how the prolonged dominance of competitive cross-modal dynamics in ASD may hinder multisensory integration and promote a detail-focused processing style.

An important challenge for future analyses will be to validate the mechanisms we propose with the present model. In particular, the default brain configuration characterized by competitive cross-modal dynamics should result in unisensory brain areas that are less activated (or more inhibited) in cases of multisensory stimulation compared to unisensory stimulation. This situation should be reversed in adults, where inhibitory cross-modal synapses have been superseded by excitatory connections. In this condition, unisensory areas should be more active in the multisensory case than in the unisensory case, because of the reciprocal excitation between sensory modalities. This prediction aligns with evidence from fMRI studies. For example, Laurienti et al. (2002) demonstrated that the ongoing activity in a unisensory cortex can be influenced by the processing of inputs from a different sensory modality, resulting in deactivation. Notably, when auditory and visual stimuli were presented together, this deactivation was no longer observed in either sensory area. These findings point to the presence of cross-modal inhibitory processes within modality-specific cortices, which appear to be context-dependent and can be selectively engaged or suppressed. It is therefore plausible that the competitive cross-sensory interactions observed in the default state of our model reflect these inhibitory dynamics, which may gradually give way to facilitatory processes as the brain matures. To address this point, we plan to acquire new fMRI data, in order to investigate these predicted developmental changes.

Finally, an important line for future research will be the implementation of the rehabilitation intervention hypothesized above for children with ASD. In particular, implementing this intervention in the form of a game could be a promising approach. Indeed, game-based interventions have several potential benefits for children with ASD. Games are highly engaging and motivating for children, making therapy sessions more enjoyable and productive (Kim et al., 2013). Moreover, games can be designed to encourage social interaction and communication, targeting specific social skills, and avoiding direct interaction with human beings that children with ASD may struggle with.

## Supplementary Material

This is a list of supplementary files associated with this preprint. Click to download.

• SupplementaryMaterials.docx

## Figures and Tables

**Fig. 9:** Model Structure. (**a**) Default configuration (simulating subjects of 6-9 years of age). A and V represent the auditory and visual regions, responsible for the first sensory processing, and implementing the input layer. They exchange direct inhibitory synapses, implementing a cross-modal competition. M is the multisensory output region. It is responsible for generating simulated reaction times (RTs) to the external stimuli. $I_{a}$ and $I_{v}$ are unimodal inhibitory areas, excited by the input layer and implementing a control mechanism via feedback inhibitory synapses and a winner-takes-all dynamics. (**b**) Adult configuration (simulating adults). Its synaptic architecture differs for i) the effectiveness of the excitatory feedforward $\left(W_{ma},W_{mv}\right)$ and inhibitory feedback $\left(L_{a},L_{v}\right)$ synapses; and ii) the now excitatory cross-modal synapses $\left(W_{av},W_{va}\right)$, implementing a cross-modal facilitation. Red lines represent excitatory connections; blue lines represent inhibitory synapses; bold lines were trained during the training phase.

**Fig. 10:** Network behavior in the auditory conditions: auditory repeat vs auditory switch. (**a**) Example of the network behavior in the auditory repeat condition (A→A). The two rectangular impulses represent the external auditory stimuli presented with an ISI of 2000 ms. Light and dark grey lines describe the activation of auditory and visual regions, respectively. Black lines represent the activity elicited in the output region in response to the stimulation. This activity is compared with the detection threshold (dashed line): the simulated RT (horizontal black bar) is calculated as the interval between the instant when the black line overcomes the threshold and the instant of stimulus presentation. (**b**) Example of the network behavior in the auditory switch condition (V→A). The two rectangular impulses represent the external auditory and visual stimuli presented with an ISI of 2000 ms. Conventions are the same adopted in the previous panel. In the repeat condition, the RT is slightly faster than the RT in unisensory switch condition. (**c**) Comparison between the simulated RTs obtained with our model and the median RTs, extracted from the real population (Crosse et al., 2022), for auditory repeat and switch conditions.

**Fig. 11:** Network behavior in the multisensory conditions: audiovisual repeat vs audiovisual switch. (**a**) Example of the network behavior in the audiovisual repeat condition (AV→AV). The two rectangular impulses represent the external audiovisual stimuli presented with an ISI of 2000 ms. Conventions are the same adopted in the previous figure. (**b**) Example of the network behavior in an audiovisual switch condition (V→AV). The two rectangular impulses represent the external visual and audiovisual stimuli presented with an ISI of 2000 ms. Conventions are the same adopted in the previous figure. In the multisensory switch condition, the RT is comparable to the RT in repeat condition. (**c**) Comparison between the simulated RTs obtained with our model and the median RTs, extracted from the real population (Crosse et al., 2022), for audiovisual repeat and switch conditions.

**Fig. 12:** Comparison between model training results and empirical developmental trajectories of TD subjects. Simulated RTs, in the repeat (upper panels) and switch (lower panels) conditions, for each sensory modality (RTs to A stimulation in the left panels; RTs to V stimulation in the central panels; RTs to AV stimulation in the right panels), are evaluated at different training epochs (solid lines) and compared with experimental data of TD children (Crosse et al., 2022) of different ages (dashed-dotted lines).

**Fig. 13:** Comparison between empirical developmental trajectories of ASD subjects and model training results with reduced plasticity. Conventions are the same adopted in the previous figure. Simulated RTs (solid lines), obtained perturbing the network with a reduced plasticity, are evaluated at different training epochs, and compared with experimental data of ASD children (Crosse et al., 2022) of different ages (dashed lines).

**Fig. 14:** Comparison between empirical developmental trajectories of ASD subjects and model training results with reduced multisensory AV exposure. Conventions are the same adopted in previous figures. Simulated RTs (solid lines), obtained perturbing the network with a reduced percentage of AV stimuli presentation, are evaluated at different training epochs, and compared with experimental data of ASD children (Crosse et al., 2022) of different ages (dashed lines).

**Fig. 15:** Effects of the highly multisensory intervention in case of reduced plasticity. (**a**) The multisensory-biased training, implemented by training the network presenting 60% of AV inputs, is applied starting from the very beginning of the training phase. The so obtained simulated RTs are sampled at different stages of the training and compared with simulated RTs obtained in case of reduced plasticity, and with the empirical RTs of TD children of the corresponding age group. The maturational delay is quickly recovered and the simulated RTs become comparable with the empirical TD ones. (**b**) The same highly multisensory training is applied starting from the second age group only. The so obtained simulated RTs are sampled at different stages of the training and compared with simulated RTs obtained in case of reduced plasticity, and with the empirical RTs of TD children of the corresponding age group. An improvement is still present, but it is very limited.

**Fig. 16:** Effects of the highly multisensory intervention in case of reduced multisensory experience. (**a**) The multisensory-biased training, implemented by training the network presenting 60% of AV inputs, is applied starting from the very beginning of the training phase. The so obtained simulated RTs are sampled at different stages of the training and compared with simulated RTs obtained in case of reduced AV exposure, and with the empirical RTs of TD children of the corresponding age group. The maturational delay is quickly recovered and the simulated RTs become comparable with the empirical TD ones. (**b**) The same highly multisensory training is applied starting from the second age group only. The so obtained simulated RTs are sampled at different stages of the training and compared with simulated RTs obtained in case of reduced AV exposure, and with the empirical RTs of TD children of the corresponding age group. An improvement is still present, but it is very limited.

## Data Availability

The data used in this study are available upon reasonable request to the corresponding author.
